# Current Perspectives of Biocontrol Agents for Management of *Fusarium verticillioides* and Its Fumonisin in Cereals—A Review

**DOI:** 10.3390/jof7090776

**Published:** 2021-09-18

**Authors:** Deepa N, Premila N. Achar, Marikunte Y. Sreenivasa

**Affiliations:** 1 Department of Studies in Microbiology, University of Mysore, Mysuru 570 006, Karnataka, India; deepanagraju@gmail.com; 2 Department of Molecular and Cellular Biology, Kennesaw State University, Kennesaw, GA 30144, USA

**Keywords:** fumonisin, biocontrol, phytopathogen, *Fusarium*, plant pathogens

## Abstract

*Fusarium verticillioides* is the most predominant fungal phytopathogen of cereals and it is posing great concern from a global perspective. The fungus is mainly associated with maize, rice, sorghum, wheat, sugarcane, banana, and asparagus and causes cob, stalk, ear, root, crown, top, and foot rot. *F. verticillioides* produces fumonisins as the major secondary metabolite along with trace levels of beauvericin, fusaric acid, fusarin C, gibberiliformin, and moniliformin. Being a potential carcinogen, fumonisins continue to receive major attention as they are common contaminants in cereals and its processed food products. The importance of elimination of *F. verticillioides* growth and its associated fumonisin from cereals cannot be overemphasized considering the significant health hazards associated with its consumption. Physical and chemical approaches have been shown to reduce fumonisin B1 concentrations among feeds and food products but have proved to be ineffective during the production process. Hence, biological control methods using microorganisms, plant extracts, antioxidants, essential oils, phenolic compounds, and other advanced technologies such as growing disease-resistant crops by applying genetic engineering, have become an effective alternative for managing *F. verticillioides* and its toxin. The different methods, challenges, and concerns regarding the biocontrol of *F. verticillioides* and production of fumonisin B1 have been addressed in the present review.

## 1. Introduction

Mycotoxigenic contamination of feeds, cereals, and cereal-based food by *Fusarium verticillioides* adversely affects the health of humans and animals leading to a decline in the economy and international trade. Developed countries uphold food suppliers’ and retailers’ high standards by implementing regulatory controls involving good agricultural management practices, hazard analysis and critical control point (HACCP), addressing food safety. In addition, the application of selected physical treatments, chemicals, and biologically based strategies substantially reduce fumonisin contamination in cereals and cereal-based products [1]. In developing countries, governing measures are poorly enforced by farming communities. Certain methods such as hand-sorting of contaminated cereals has been practiced and seem to be partially effective, remaining as the last line of defense in reducing fumonisin and mycotoxin exposure [2].

Studies have shown that consumption of mycotoxin-contaminated cereals can affect the lungs, liver and kidneys in animals and cause wounds, skin lesions, and even lead to cancer in humans [3,4]. In horses, being fed with naturally contaminated corn, corn screenings, and corn-based feeds or intravenous injection of fumonisin leads to leukoencephalomalacia (LEM) [5]. Pulmonary edema syndrome (PES) and hydrothorax were observed in pigs on consumption of fumonisin-B1-contaminated corn screenings and through intravenous injections of fumonisin, respectively [6]. Voss et al. [7] reported that hydrolyzed FB1 (HFB1) interferes with sphingolipid metabolism without causing any neural tube defects in a mouse model. When the blood–brain barrier was permeated in young carp, neurotoxicity due to FB1 was reported [8]. On consumption of the corn associated with fumonisins or contaminated with *F. verticillioides*, reports indicate large numbers of cases of esophageal cancer, among humans, in Transkei, South Africa [9], Northern Italy [10], Linxhian, China [11], the south-eastern United States, and Golestan, Iran [12]. The International Agency for Research on Cancer (IARC) characterized FB1 as a possible group 2B carcinogen, which can cause toxicity in humans and several animals like rats, horse, mice, and rabbits [13].

## 2. Worldwide Association of *F. verticillioides*

*Fusarium verticillioides* distribution is ubiquitous, mainly associated with maize [1,14], rice [15,16,17], sugarcane [18], wheat [19], banana [20], asparagus [21,22], and sorghum [23]. Rocha et al. [24] screened maize grain samples from Brazil and documented nearly 96% frequency of *F. verticillioides.* The highest incidence of *F. verticillioides* was reported in poultry and animal feed made up of wheat bran and maize pellets [25]. Among the 135 cereal samples collected from southern India, 69 were associated with *Fusarium* contamination, among which 51 samples showed *F. verticillioides* [26]. In southern Europe, Italy, and Iran, *F. verticillioides* was the predominant species associated with maize grain samples [27,28,29]. Among 103 *Fusarium* species screened from the cereal samples collected from Karnataka, India, 64 isolates were found to be fumonisin-producing *F.* *verticillioides* [30].

A major focus by scientists across the globe is on mycotoxigenic fungi since they are global contaminants of cereals and cereal-based food products [3]. Mycotoxins such as fumonisin B produced by *F. verticillioides* in cereals, is categorized into FB1, FB2, FB3, and FB4, based on the structure and hydroxyl group [3]. Fumonisin B1, the most predominant and toxic mycotoxin accounts for 70% of the total fumonisins and receives worldwide attention compared to FB2, FB3, and FB4 [31] (Table 1). FB1 toxicity seems to be complex resulting in disruption of de novo biosynthesis of ceramide-deregulating sphingolipid complex [32]. Exposure to FB1 toxin among humans and animals leads to the accumulation of spingoid bases, increased phosphate adducts, and reduced ceramide concentrations, resulting in apoptosis, cytotoxicity, cell proliferation [33], neural tube defects, hepatocarcinoma, carcinogenicity, and DNA damage [34].

The fumonisin-producing *F. verticillioides* strains were initially confirmed through various PCR methods [35,36], such as multiplex PCR and nested PCR which showed direct association of fumonisin with cereals, pure cultures, and plant parts. Both species-specific and fumonisin-specific genes were detected in a single test run in case of multiplex PCR [37]. While nested PCR involved two test runs, in which the first test run with species-specific primer product, served as DNA for the second test run with different primer for fumonisin [38]. Chromatographic techniques, such as high-performance liquid chromatography [39] and liquid chromatography mass spectrometry [40] were used to quantify FB1 toxin associated with cereal samples. According to the Food and Agriculture Organization (FAO) and the World Health Organization (WHO), tolerable maximum intake for fumonisins has been set as 2 µg/Kg·bw/day based on lack of any observed adverse effects for nephrotoxicity in male rats [41].

## 3. Management of *F. verticillioides*

Many studies have been reported on various prevention strategies and pre-harvest control methods such as disease-resistant varieties, biocontrol agents such as microorganisms, and plant extracts against growth and toxin production by *Fusarium* species [42,43]. Several researchers examined post-harvest removal of fumonisin from food commodities by physical, chemical, and modest biological control methods [44,45]. Proper agricultural practices need to be maintained during pre-harvest and post-harvest to minimize the growth of *F. verticillioides* and its toxin production in cereals. Fumonisins are managed by prevention of *F. verticillioides* infection, in addition to the monitoring of the contaminants, and their detoxification [46].

## 4. Physical Methods for Management of *F. verticillioides*

Physical methods comprise of the separation of damaged or contaminated crops from healthy ones including methods like sorting, sieve cleaning, density segregation, washing, de-hulling, and steeping. Drying of grains to lower the moisture content is one of the preliminary and safest method against growth of molds and grain can be safely stored for a longer duration [47]. In addition, separation of infected and physically damaged grains from the healthy ones is an efficient and feasible method of reducing mycotoxin contamination [48]. Heating, another physical factor, which is a procedure carried out during various food processing at temperatures >150 °C, degrades the stability of fumonisin, and hence, it is considered as a good method for the removal of mycotoxin through leaching [49]. A novel physical method, of recent interest, is use of non-thermal techniques such as cold plasma for fungal and mycotoxin removal. Cold plasma is an ionized gas containing partially ionized atoms with zero net charge [50]. Another emerging non-thermal technique used for removal of mycotoxins, is the photocatalytic detoxification of mycotoxins in food. This method comprises of chemical reactions induced by absorption of photons by a solid photocatalyst, resulting in oxidation or reduction reactions on the surface of photocatalytic materials that produce free radicals which interact with contaminants such as fumonisin, and help to degrade or reduce the toxin [51].

Furthermore, irradiation such as by X-rays, gamma rays, or accelerated electrons is reported as an alternative method to control mycotoxin-producing molds in certain food products [52]. Maize and sorghum grain samples with a weight of 250 g were exposed to 2.5, 5.0, 7.5, and 10.0 kGy of gamma irradiation for evaluation of *F. verticillioides* incidence (%) and fumonisin levels at regular intervals of 0, 30, 60, and 90 days of storage [53]. Results revealed that on day 0, the incidence of *Fusarium* species was 48 and 38%, respectively, in maize and sorghum samples and there was a gradual decrease in the incidence of *Fusarium* species at 2.5 and 5.0 kGy doses of gamma irradiation after 30, 60, and 90 days storage. Deepthi et al. [54] reported that ionizing radiation at 7.5 kGy was lethal for *Fusarium* species growth and its FB1 production. In addition, they also observed that γ-radiation, above 7.5 kGy, effectively prevented *Fusarium* growth and minimized the exposure of animals and humans to fumonisin. The FAO, IAEA, and WHO stated that irradiated foods with less than 10 kGy doses are considered to be safe and nutritionally adequate when produced under established good manufacturing practices [55].

## 5. Chemical Methods for Management of *F. verticillioides*

Chemical methods to decontaminate fumonisin in maize and maize products have also been previously reported. Fumonisin is a stable molecule; hence, its destruction is challenging [56]. Munkvold et al. [56] reported a significant reduction of FB1 of up to 95% by treating contaminated maize seeds with Ca(OH)_2_. Lu et al. [57] reported fumonisin degradation using sodium bicarbonate and hydrogen peroxide. Fructose, in the presence of a primary amine and water (pH > 7), removes the preliminary amine group from fumonisin through non-enzymatic browning and has been reported to cause a drastic reduction of FB1 in maize grains and eventually in rat models. In this study, it was reported that removal of the amine group caused structural changes in fumonisin and in its ability to cause cancer in rat models [58]. Combination of ammonization and high temperature leads to fumonisin detoxification [59]. Use of chemicals methods, seem to decrease fumonisin levels significantly, compared to physical methods such as air drying of cereals or grains. This observation was reported by Scott [60] who found that treatment of cereals or grains with 2% ammonium hydroxide at 50 °C decreases fumonisin concentration to89%, compared to four days of air drying which reduced the toxin by only 32%.

Consumption of maize and maize-based products, in large amounts, may result in high risk of exposure to fumonisin. Previous studies have reported that nixtamalization (alkaline cooking) of maize grains results in hydrolyzation of fumonisin and lowers its toxicity [61,62]. A study in Central America, reported that the nixtamalization of maize grains during the production of tortilla, significantly reduced the fumonisin concentration. HFB1 (hydrolyzed fumonisin) was detected in staple food such as commercial masa and tortilla chips [63]. Another study documented a 100% reduction of fumonisin in contaminated maize by eradication of the mutagenic potential of maize extracts, when subjected to a modified nixtamalization procedure [64]. On the contrary, Voss et al. [65], suggested that chemical procedures such as nixtamalization, reduced only the detectable fumonisin levels but did not result in toxicity reduction. Most importantly, detoxification methods should be capable of removing all traces of active toxin, must not leave any hazardous chemical residues in the treated samples, and above all, should not compromise the nutritional value of the food [66].

## 6. Limitations of Physical and Chemical Methods

Many of the physical and chemical methods used to minimize fumonisin B1 concentrations in cereals or cereal-based foods have proved to be either ineffective or difficult to practice in the food production process [67,68]. The detoxification methods of fumonisin, must be cost effective, simple, and easily applied by farmers. While physical methods were found to have low efficacy and less specificity, chemical methods were found to be expensive, and affect the quality of food products by producing toxic derivatives and decreasing the nutritional value of the food [48,69]. Certain chemical compounds used as antifungal agents were not biodegradable and resulted in contaminated water and soil [70,71]. Prolonged use of chemical treatments during cultivation of grains and cereals, has enabled the hosts to establish resistance against the fungal pathogens. Increased demand for the use of chemicals in agriculture to combat pathogens has escalated toxic residues in food crops [72]. Hence, there is a great demand for the alternative and safe methods to control *F. verticillioides* and fumonisin contamination in cereals.

## 7. Management of *F. verticillioides* by Natural Clay

Natural clay adsorbents have been considered as Generally Recognized as Safe (GRAS) by the Food and Drug Administration, USA [73]. Natural clay adsorbs mycotoxins in food and its products by detoxifying the contaminated food during processing stages [74]. Montmorillonite clay is more significant than other clay minerals due to its large surface area and molecular structure that increases its adsorbing ability when wet. Montmorillonite clay at a low dose of 1.5 g and a high dose of 3 g/per day, through adsorption, effectively reduces FB1 in aqueous solution in vitro, and in human and animal models in vivo respectively. The adsorption is saturable and occurs largely within the interlaminar regions of the clay [75]. In addition, it was reported that a combination of clay and modified yeast cell extracts enhances adsorption of multiple mycotoxins [76]. Natural clay has also been demonstrated to be suitable for commercial use by the Selection Committee on GRAS substances (SCOGS) since they could be applied effectively and economically in the food and feed industries [77]. However, application of clay minerals often requires high levels to be included into animal feed; interaction of natural clays with food and gut-based nutrients remains unclear, and the possibility of accumulation of dioxin in animals remains a concern [1].

## 8. Biological Control of *F. verticillioides*

**Figure 1:**
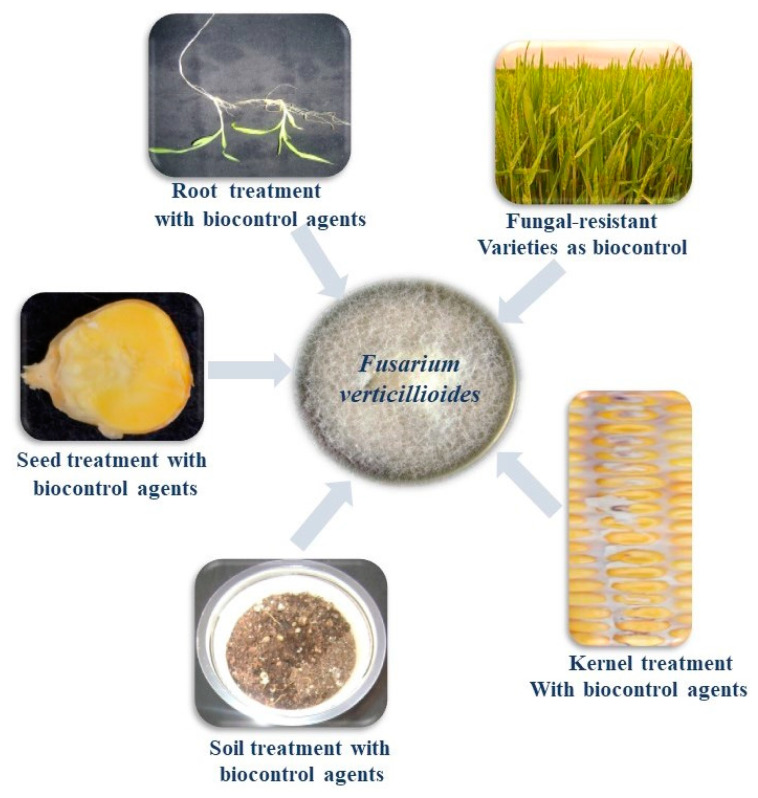
Treatment of plant parts, soil and use of resistant varieties, as biocontrol methods against *F. verticillioides*.

**Figure 2:**
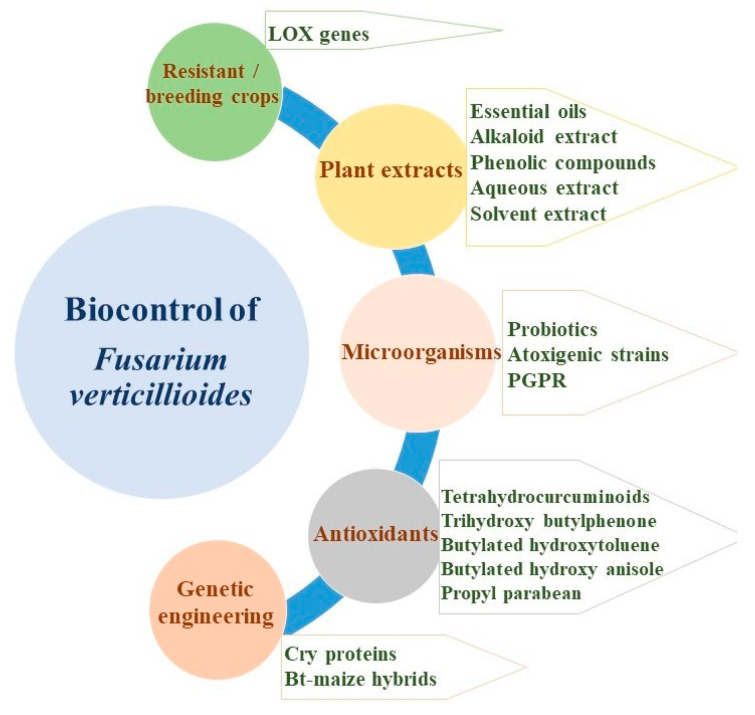
Use of biological control agents against *F. verticillioides.* PGPR, plant-growth-promoting rhizobacteria; LOX genes, lysyl oxidase genes; Bt-maize, *Bacillus thuringiensis* maize.

Biological control methods can be employed to minimize the contamination of fumonisin produced by *F. verticillioides*. According to Alberts et al. [1], simple pest control strategies were found to have a positive impact on food security and safety, especially in the rural areas where maize is a staple diet. Simple and effective biological strategies have attracted the attention of farmers throughout the world [78]. *F. verticillioides* being a phytopathogen, is mainly associated with cereals such as maize and sorghum and is largely responsible for the economic losses worldwide [79]. Currently, minimizing the soil-borne pathogens by application of synthetic fungicides or chemical pesticides has been challenged by biological approaches that play a major role in sustainable agriculture. Such biocontrol methods can be effectively adopted by binding the biocontrol agents through plant parts such as roots and seeds, and/or the soil against the target pathogen (Figure 1). Probiotics, non-toxigenic strains of fungi, plant-growth-promoting rhizobacteria, antioxidants, plant extracts, genetic engineering, and disease-resistant crop varieties have been used as biological management strategies against fumonisin-producing *F. verticillioides* (Figure 2).

### 8.1. Microorganisms as Biocontrol Agents

The application of microorganisms that have the ability to colonize infected plant parts under certain appropriate and compatible environmental conditions, has become the recent trend in minimizing the growth of pathogens and toxin production [72,80].

#### 8.1.1. Bacteria as Biocontrol Agents

The American Food and Drug Administration considers *Bacillus subtilis* as Generally Regarded as Safe (GRAS), since the organism can be easily cultured and genetically manipulated as it appropriately fits into the industrial processes. *B. subtilis*, an endophytic bacterium, and an ecological homologue with *F. verticillioides* in maize, reduced nearly 50% of FB1 accumulation during vertical transmission which spreads from plant to cob [44]. Another report indicated that *B. mojavensis*, the fusaric-acid-resistant mutant strain, the wild type, showed biocontrol potential against *F. verticillioides* [81]. Furthermore, *F. verticillioides* in vitro, has also shown its antagonistic properties against *B. amyloliquefaciens* without causing any changes in the rhizospheric region [82]. Microorganisms such as *Exophiala spinifera* (ATCC 74269), *Sphingopyxis macrogoltabida* (MTA 144), *Bacterium* so (ATCC 55552), and *Rhinocladiella atrovirens* (ATCC 74270) are capable of minimizing the production of FB1 in *F. verticillioides* [83,84,85,86,87,88] (Table 2).

It was reported that in greenhouse conditions, *B. amyloliquefaciens* and *Enterobacter hormacchei*, at a concentration of 10^9^ CFU mL^−1^ reduced the infection of maize grains against *F. verticillioides* and fumonisin production in the soil, thereby improving the quality of maize grains [82]. In greenhouse trials, root applications of *B. subtilis* (10^8^ and 10^7^ CFU/mL), against *F. verticillioides* has become the potential biocontrol method due to the ability of *B. subtilis* to reduce the endorhizosphere and rhizoplane colonization with the pathogen [89]. Root infection of maize seedlings by *F. verticillioides* was minimized followed by *B. amyloliquefaciens* treatments, and it was reported as an effective preventive measure against horizontal transmission (transmission between neighboring plants) of pathogens, without affecting the normal plant growth [90] (Table 3).

**Table 2:** In vitro effect of biocontrol agents on growth (%) and development of toxigenic *F. verticillioides*.

Serial Number	Test Organism	Methods Used for Screening	Targeted Feature of *F. verticillioides*	Percent of Reduction	Reference
Microorganisms					
1.	*Bacillus subtilis*	Antifungal activity	Fungal growth	50%	[91]
2.	*Lactobacillus rhamnococcus*	Antifungal activity	Mold growth	78–92%	[90,92]
3.	*Saccharomyces cerevisiae*	Antifungal activity	Mold growth and FB1	77–89%	
4.	*Pediococcus pentosaceus*	Antifungal activity and spectrum	Zone of inhibition	89%	[93]
5.	*Enterococcus casseliflavus M4A*	Antifungal activity	Fungal growth	100%	[94]
6.	*Trichoderma viridae*	Antifungal activity	Zone of inhibition	90%	[70,95,96]
7.	*Trichoderma harzianum*	Antifungal activity	Zone of inhibition	< 70%	
8.	*Pseudomonas solanaceacum*	Antifungal activity	Zone of inhibition	70%	[81]
9.	Mixture of *E. cloacae* and *M. oleovorans*; mixture of *P. solanaceacum* and *B. subtilis*	Antibiosis	Root colonization	50%	[87]
**Antioxidants**					
10.	Butylated hydroxyanisole (BHA)	Water activity	Mycelial growth	94–98%	[68]
11.	Propylparabean (PP)			100%	
12.	Trihydroxybutyrophenone (THBP)			>85%	[97]
13.	Butylated hydroxytoluene (BHT)			95%	
14.	Tetrahydrocurcuminoids (THC)	Antifungal activity	Fungal conidial growth	100%	[98]
**Phenolic Compounds and Plant Extracts**					
15.	Geranial, eugenol, and singerone from *Zingiber officinale*	Poisoned food technique	mycelial growth	100%	[99]
16.	Carvacrol, eugenol, 2-hexanal from oregano, thyme, cinnamon, clove, fruits, and vegetables	Antifungal activity	Conidial germination and mycelial growth	37–97%	[100]
17.	Chitin-binding protein from *Ginko biloba*,	Antifungal activity	Mycelial growth	>50%	[101]
18.	Vanillic acid and caffeic acid	Antifungal activity	Fungal growth	80% 100%	[102,103]
19.	Chlorophorin, iroko, maakianin, and ferulic acid	Agar-well diffusion and HPLC	Fungal growth and FB1	88–94%	[103]
20.	Flavonoids, phenolic acid, and terpine-rich ethanol extracts from *Equisetum arvense* and *Stevia rebaudiana*	Antifungal activity	Fungal growth	79%	[104]
21.	Aqueous extract, methanol extract, and alkaloid extract from *Prosopis juliflora*	Poisoned food technique	Mycelial growth	100%	[105]
22.	1,8-Cineole from *Rosnainum officinalis*	Antifungal activity	Conidial production	53.48%	[106]
23.	Eugenol, methyl eugenol, and tumerone from *Syzygium aromaticum, Pimenta dioica, and C. longa*		Fungal growth	40–80%	

#### 8.1.2. Probiotics as Biocontrol Agent

Furthermore, lactic acid bacteria have been applied as a safe biocontrol agent to minimize the growth and production of *F. verticillioides* and fumonisin production. Certain in vitro studies have shown the inhibitory potential of *Saccharomyces cerevisiae* and *Lactobacillus rhamnous* against *F. verticillioides* growth [92]. Reports of in vivo studies indicated elimination of FB1 content in mature mice administered with the biocontrol agent, *S. cerevisiae* [93]. In another report, *S. cerevisiae* as a biocontrol agent, was observed to control the growth of *F. verticillioides* and FB1 production in cereals [87]. *Pediococcus pentosaceus*, with a GRAS status, is widely used as a biocontrol organism in food fermentation and ensilage quality improvement. Additionally, the supernatant of *P. pentosaceus* exhibited antifungal activity thereby inhibiting growth of both *F. proliferatum* and *F. verticillioides* [107]. Recently *Enterococcus casseliflavus* M4A strain was reported to be a promising tool for biocontrol of *F. verticillioides* in storage maize grain silos. Combination of volatile organic compounds diacetyl and acetic acid produced by *Enterococcus casseliflavus* M4A strain completely inhibited *F. verticillioides* growth and acetoin significantly (88.75%) reduced FB1 biosynthesis [94] (Table 2 and Table 4).

#### 8.1.3. Fungi as Biocontrol Agents

Among the fungi, *Trichoderma* species are considered as an effective biocontrol agent against *F. verticillioides* due to their ability to produce extracellular lytic enzymes [47]. Harmosa et al. conducted in vitro and in planta studies in maize and suggested by GRAS status that, *T. harzianum and T. viridae*, effectively reduced the growth of *F. verticillioides* and its fumonisin production by producing extracellular enzymes, volatile compounds, and antibiotics [95,108] (Table 2 and Table 3). *Trichoderma* species were also applied as postharvest biocontrol agents, which reduced the colonization of *F. verticillioides* and its toxin accumulation in corn during storage [96] (Table 2).

#### 8.1.4. PGPR as Biocontrol Agents

Naturally occurring bacterial species *Azotobacter* and *Arthrobacter* were found to be prevalent and predominant mainly in the rhizoplane and endorhizosphere of maize-growing areas [91]. *Enterobacter cloacae* has also been reported as a noteworthy biocontrol agent against *F. verticillioides* during root colonization of maize crop [109]. Biological control potentials of bacteria in mixed cultures of *E. cloacae, Microbacterium eoleovorans, P. solanacearum*, and *B. subtilis* have shown synergistic activities by prevention and reduction of vertical transmission and colonization of roots by *F. verticillioides* in maize seed [87] (Table 2). Other bacterial species, such as *Pseudomonas solanacearum, Azotobacter armeniacus*, and *Arthrobacter globiformis* and rhizobacterial strains of *Bacillus* species were all found to exhibit potent in vitro antifungal activities as seed inoculants against *F. verticillioides*, thereby reducing its growth and FB1 production in the endorhizosphere and rhizoplane region of the maize root [68,91] (Table 2).

#### 8.1.5. Mycoviruses as Biocontrol Agents

Currently, the application of mycoviruses as biocontrol agents both in vitro and in vivo is in great demand. Mycoviruses induce hypovirulence among host fungi as they lack extracellular transmission routes [110]. Three different mycoviruses FgV1, FgV2, and FgV3 induced hypovirulence and caused latent infections involving the role of RNAi among *Fusarium* species [111].

#### 8.1.6. Non-Toxigenic Strains as Biocontrol Agents

Other strategies have also been used for biological control against *Fusarium* species. Non-pathogenic *Fusarium* strains have been moderately applied as biocontrol agents in suppressing the growth of toxigenic strains *F. proliferatum* and *F. verticillioides* in maize [112]. The gene silencing technique has been applied as a biocontrol strategy by deleting ZFR1 in *F. verticillioides.* This method affects fumonisin biosynthesis and regulates the sugar transporter genes during the formation of kernel colonization, resulting in minimized growth of maize endosperm; development of the endosperm plays a major role in biosynthesis of fumonisin in maize grain [113].

### 8.2. Antioxidants as Biocontrol Agents

Antioxidants, namely propylparaben (PP) and butylated hydroxyanisole (BHA), are considered GRAS by the FDA, and they are used as preservatives in certain food and cosmetic industries [114]. Under respective in vitro conditions, both BHA and PP were observed to suppress the growth of *F. verticillioides* and *F. proliferatum* [115], indicating their potential as biological control entities. Similarly, in a dose-response study, a 77% reduction of *F. verticillioides* was reported with 10–100-fold doses of BHA/PP (500 µg/g) at water activity (0.95) for a period of 28 days [116]. Reynoso et al. [117] reported a higher percentage of reduction in fumonisin production during a combinational treatment of BHA and PP over other antioxidants, butylated hydroxytoluene (BHT) and trihydroxybutyrophenone (THBP) [97]. Another antioxidant, tetrahydrocurcuminoids (THC), and its related molecules extracted from non-toxic plant, *Curcoma longa*, was also found to suppress FB1 production in vitro [98]. Biosynthesis of FB1 from *F. verticillioides* was strongly inhibited by 3,6,7-trihydroxy-α-tetralone (TT) extracted from *Phoma moricola* at 200 µg/mL concentration. TT is strongly active against the enzyme polyketide synthase as antimycotoxin, which could be explored as an eco-friendly method for managing mycotoxin contamination in food and feed stuffs [118] (Table 2 and Table 4).

**Table 4:** Effect of biocontrol agents on the reduction of fumonisin B1 production by *F. verticillioides*.

Serial Number	Biocontrol Agents	Detection Method	Percent of FB1 Reduction	Level of Study	Reference
Microorganisms					
1.	*Bacillus subtilis*	HPLC	50%	In vitro	[87]
2.	*Bacillus amyloliquifaciens*	HPLC	>70%	Field study	[82,89]
3.	*Microbacterium oleovarans*	HPLC		Field study	
4.	*Enterobacter hormacchei*	HPLC		Field study	
5.	*Lactobacillus rhamnococcus*	HPLC	78–92%	In vitro	[90,92]
6.	*Saccharomyces cerevisiae*	HPLC	77–89%	In vitro	
7.	*Pseudomonas solanaceacum*	HPLC	70–100%	In vitro	[68,81,87]
8.	Mixture of *E. cloacae/M. oleovorans;* mixture of *P. solanaceacum/B. subtilis*	HPLC	100%	Field study	[91]
**Antioxidants**					
9.	Butylated hydroxyanisole (BHA)	HPLC	100%	In vitro	[68,117]
10.	Propylparabean (PP)		94–98%		
11.	Trihydroxybutyrophenone (THBP)		94–98%		[97]
12.	Butylated hydroxytoluene (BHT)				
13.	3,6,7-trihydroxy-α-tetralone (TT)	HPLC	>95%	In vitro	[118]
**Phenolic Compounds and Plant Extracts**					
14.	Ferulic acid	HPLC	98–100%	In vitro	[103]
15.	Vanillic acid, and caffeic acid	HPLC	98–100%	In vitro	[102]
16.	Acetonin	HPLC	88.75%	In vitro	[94]
17.	Acetone extract	HPLC	96%	In vitro	[119]

### 8.3. Plant Extracts as Biocontrol Agents

Antifungal assays of plant extracts, and phenolic compounds from plants have been studied for long time, and these compounds have been identified as inhibiting the growth of *F. verticillioides* and suppressing fumonisin production. Aqueous and methanol extracts of the plant *Prosopis juliflora* inhibited the growth of *F. verticillioides* by 50% and 65% at 400 µg mL^−1^, respectively, whereas alkaloid extract of *P. juliflora* completely inhibited the growth of *F. verticillioides* at 300 µg mL^−1^ [105]. Recently, *Tagetes erecta* methanol extract from leaves, flowers, and roots of the plant were found to inhibit *F. verticillioides* growth by more than 65% after 7 days of incubation [108] Combination of *Combretum erythrophyllum* and *Quercus acutissima* acetone extract exhibited 96% inhibition against *F. verticillioides* growth [119]. In addition, *F. verticillioides* growth was also inhibited by 34% with highly potent betel leaf extract at 1000 ppm concentration [120,121]. In another interesting report, incidence of *F. verticillioides* was lowered from 40% to 25% by storing maize in bamboo granaries instead of on cement floors; the bamboo granaries served as a biocontrol agent [122]. Phenolic compounds, namely, thymol, carvacrol, and eugenol, were identified to be the most active antifumonisin compounds among 10 natural phenolic compounds tested [120]. A chitin-binding protein from *Ginko biloba* and a polygalacturonase-inhibiting protein from *Arabidopsis thaliana*, inhibited growth of fumonisin-producing *F. verticillioides* [101,123]. Similarly, phenolic compounds such as caffeic and vanillic acid were observed to decrease the growth *F. proliferatum* and *F. verticillioides* and FB1 production in maize [124]. The authors, in their in vitro studies, observed an increase in concentration of phenolic compounds, such as caffeic acid and vanillic acid, which completely inhibited the growth of fungus and FB1 production; however, the growth inhibition (%) of vanillic acid was more effective than caffeic acid [102]. In addition to vanillic acid and caffeic acid, iroko, chlorophorin, maakianin, and ferulic have also been reported to inhibit the growth of *F. verticillioides* and the biosynthesis of fumonisin B1 [103]. Non-toxic plants extract such as flavonoids, phenolic acid, and terpene-rich ethanol extracts from *Stevia rebaudiana* (candy leaf) and *Equisetum arvense* (horsetail) inhibited the conidial growth of *F. verticillioides;* however, they were less effective against the fumonisin production. Extracts of *Gynostemma pentaphyllum* were observed to inhibit only the growth of *F. verticillioides* [104,125]. Recently, for the first time, an experiment has been conducted on stalk rot and reported that synergistic activity of betel leaf extract with *B. subtilis* TM3 formulation resulted in 20% inhibition against stem rot disease and 13.37% against cob rot disease in maize plants [126] (Table 3 and Table 4).

### 8.4. Plant-Based Essential Oils as Biocontrol Agents

Plant-based essential oils and their active ingredients play an important role in direct and indirect plant defenses against pathogens and serve as antimicrobial compounds. Essential oils extracted from anise and thyme have been reported to cause complete inhibition of *F. verticillioides*. The growth reduction of *F. verticillioides* was reported up to 79% by caraway and 86% by fennel, and more than 60% inhibition was reported by spearmint, marigold, hazanbul, onynum, basil, and chamomile essential oils at concentration ≤500 ppm [127]. Essential oils with certain constituents were extracted from aromatic plants (*Aloysia polystachya*, *Origanum vulgane, Mentha piperita*, and *Aloysia triphylla)* and these oils inhibited growth and fumonisin production in *F. verticillioides* [128]. Essential oils of lemon grass, cinnamon leaf, clove, palmarosa, and oregano have also been shown to inhibit mycelial growth of *F. verticillioides*, *F. proliferatum*, and *F. gramineareum* under different temperature (20–30 °C) and water activity (0.95–0.995) conditions [129]. Furthermore, essential oils from neem, cymbopogon, eucalyptus, clove, peppermint, and cedar wood were screened for their efficacy against *Fusarium* species in maize and sorghum, and of all the oils tested, citronella from *Cymbopogon nardus*, at a concentration of 500 ppm, inhibited the growth of nine species of *Fusarium* [130]. Essential oils such as geranial, eugenol, and singerone (oleoresins) extracted from *Zingiber officinale* exhibited antifungal potential and were reported to be effective against the growth of *F. verticillioides* [131]. Carvacrol, eugenol, and 2-hexanal (extracted from oregano, thyme, cinnamon, clove, fruits, and vegetables), effectively inhibited the mycelial growth and conidial germination of *F. verticillioides* in maize kernels [99]. Chemical compounds extracted from essential oils, namely eugenol from *Syzygium aromaticum*, methyl eugenol from *Pimenta dioica*, and α-tumerone and β-tumerone from *Curcuma longa* inhibited *F. verticillioides* growth by 88.70%, 53.09, 44.20%, and 70.67%, respectively, whereas 1,8-cineole extracted from *Rosanium officinalis* inhibited conidial production of *F. verticillioides* by 53.48% [94,100,106]. Recently, essential oils extracted from *Anacyclus valentinus*, *Carum carvil*, cinnamon, *Cumin cyminum*, *Cymbopogon nardus, Foeniculum vulgare, Ocimum basilicum*, and *Thymus capitatus* inhibited growth of *F. verticillioides* from 75 to 92% by micro and macro dilution methods [132,133,134,135,136,137,138] (Table 3 and Table 4). Essential oils and their components are important because of their low cost, availability, and wide range of biological activities. Antibacterial and antioxidant abilities of essential oils are well documented but studies on antifungal and antimycotoxigenic abilities of essential oils are still limited [139].

### 8.5. Resistant Crops via Breeding as Biocontrol Methods

The resistant crops grown through genetic engineering and breeding techniques have been designed primarily for avoiding contamination by mycotoxigenic fungi, insect invasion, and mycotoxin detoxification in planta by using gene manipulation studies [140,141,142]. Transcriptional changes by inoculating *F. verticillioides* among susceptible and resistant genotypes in maize is done by next-generation RNA sequencing [143]. This method provides an important genomic resource in developing disease-resistant maize genotypes [143]. Information on biochemical and molecular methods, elucidating concepts of natural resistance in crops, has become important for further progress in development of resistance to infection by *Fusarium* and insect infestation in crops [144]. Infection of *F. verticillioides* in maize indicates up-regulation of genes encoding various ranges of proteins associated with virulence or susceptibility, resistant maize lines, defense, rescue, permatin proteins, pathogenesis proteins, proteins scrambled in detoxification response, proteinase inhibitors, and heat shock proteins [145,146]. Maize lipoxygenase (ZmLOX) derivative of oxylipins has been identified as contributing to the adaptable plant defense against pathogens. Metabolic activity of lipoxygenase derivatives, including up-regulation of ZmLOX5, LOX genes, and ZmLOX12, has been identified as more specific derivatives during the host–pathogen interactions of maize and *F. verticillioides* [147].

### 8.6. Genetic Engineering as Tools for Biocontrol

Genetic engineering tools improve commercially acceptable crops by certain mechanisms, such as natural, fungal, and insect resistance [142]. Cry proteins from *Bacillus thuringiensis* (Bt), isolated from Bt maize, were genetically modified and considered as safe to consumers. These cry proteins were highly effective in reducing the level of fumonisin production and insect damage in maize when compared with non-Bt hybrids [148]. Corn borers harm the ear tissue and stalk of maize, stimulating the spore germination of *F. verticillioides*, followed by increased fumonisin production. The vital association of insect damage and total fumonisin level in maize resulted in ear and kernel rot [149]. Similarly, the results of in planta trials in USA and Europe, reported lower fumonisin levels in Bt maize hybrids. Furthermore, such hybrids have been reported to increase the percentage of yield and are environmentally friendly and fit for human and animal consumption as per the World Health Organization (WHO) and the Environmental Protection Agency (EPA) [150]. By lowering fumonisin and aflatoxin contamination in the USA, the annual benefit by Bt maize was reported as USD 23 million [150]. The use of hybrids has become an important tool in developing countries. Bt plants reduce fumonisin production in maize during seasons when the European corn borer (*Ostrinia nubilalis* Hübner) dominates in the field; however, it is not the case when the corn earthworm (*Helicoverpa zea* Boddie) dominates the field [149].

### 8.7. Commercially Available Products as Biocontrol Agents

Certain commercial products, other than Bt have been used as biological control agents against *Fusarium* species: Fusaclean and Biofox C from atoxigenic *F. oxysporum* strain against *F. verticillioides* in vegetables; Epic and Kodiak from *B. subtilis*; Intercept from *Pseudomonas cepacian*; Mycostop from *Streptomyces griseoviridis*; T-22G, T-22HB, and Biofungus from *Trichoderma harzianum;* Blue Circle and Deny from *Burkholderia cepacian*; Cedom and Cerall from *Pseudomonas chlororaphis* [1]; Novasil and Nevalite from clay material [75]; and Fumzyme from *S. macrogoltabida* [88]. Although these are biological control agents proven to be environmentally safe, contamination of cereals by *F. verticillioides* and production of fumonisin continue to be a global threat.

## 9. Conclusions

Additional research data on *F. verticillioides*, and exposure and safety evaluations of fumonisin are needed to evaluate the potential toxicity of this toxin and its byproducts. Further research on the safety of physical, chemical, and biological decontamination are needed, and specific strategies that combine an integrated decontamination approach must be developed to remove the fumonisin content from cereals and cereal-based foods to the greatest possible extent [151]. Research has been effectively carried out across a wide area to reduce the growth of *Fusarium* species and fumonisin production during pre-harvest and post-harvest stages by practicing natural and biological methods, including plant materials, minerals, and microorganisms.

Usage of physical methods, even though they seem to be acceptable practices and cause limited change in the properties of the commodity, still seems to be impractical and limiting for large-scale industries as they are time-consuming and expensive. While several of the chemical treatments are affordable and effective against mycotoxins, their use is still banned by the European Union (EU) in food processing, since they can pose certain health risks due to possible toxic byproducts generated.

From our review, it appears that application of biological methods in lowering the fumonisin production by *F. verticillioides*, supersedes other measures we have listed, though with a few disadvantages. Microorganisms such as bacteria, fungi, PGPR, probiotics, and atoxigenic strains, even though they are beneficial in minimizing the fumonisin content and cost effective, may become harmful at some stage in their growth and development. Similarly, plant-based natural products such as essential oils, antioxidants, and plant extracts, are derived using certain chemicals, and have been used as a biocontrol agent. Certain essential oils extracted from plants, have shown a wide range of antifungal activities, including minimizing production of fumonisin content by *F. verticillioides* however, it has been reported that the structure obtained after extracting the essential oil appears to be toxic. On the other hand, first-line defense methods, such as development of resistant varieties and application of genetic engineering methods to minimize the production of mycotoxins by fungi, have recently been used, however, only in crops of economic importance.

Resistance to fungal infection by genetic engineering does not seem to be a long-term solution since such varieties are not affordable by most of our farmers. Biological methods, though they are inexpensive and cause no harm to the environment, are time-consuming and impractical in some set-ups. In conclusion, perhaps, additional extensive in vitro and in vivo studies and much more international collaborative research must be initiated on *F. verticillioides* and production of fumonisin B1 to add data to the existing knowledge on control measures for this pathogen in the field, at storage, and in the processing period. Such collaboration may lead to a total global control of this fungus and eradication of this carcinogenic toxin in our food chain.

## Figures and Tables

**Table 1:** Diseases and toxins produced by *F. verticillioides* in different cereal crops.

Serial Number	Crop	Disease	Toxins
1.	Corn/Maize (*Zea mays*)	*Fusarium* ear rot, stalk rot, kernel rot, root rot, seed rot, seedling blight, seedling root rot	FB1, FB2, and FB3
2.	Cultivated wild rice (*Zizania palustris*)	Scab	FB1, FB2
3.	Oats (*Avens sativa*)	*Fusarium* foot rot, snow mold, seedling blight, head blight	FB1, FB2
4.	Pearl millet (*Pennisetum glauccum*)	Top rot	FB2, FB1
5.	Rice (*Oryza sativa*)	Seedling blight, water mold, root rot, pecky rice (kernel spotting)	FB1, FB2, FB3
6.	Sorghum (*Sorghum bicolor*)	Damping off and seed rot, *Fusarium* wilt head blight, root and stalk rot, twisted top, seedling blight, seed rot	FB2, FB1
7.	Sugarcane (*Saccharum* spp.)	*Fusarium* stem rot, pokkah baeng, wilt;	FB1, FB2
8.	Wheat (*Triticum* spp.)	Black point (kernel smudge), head blight (scab), root, crown, and foot rot, pink snow mold;	FB1, FB2

Source: www.apsnet.org/online/common/search.asp accessed on 1 August 2018.

**Table 3:** In planta effect of bacteria as biocontrol agents against *F. verticillioides* in maize.

Serial Number	Plant Parts Treated with Biocontrol Agents	Test Organisms	Targeted Feature	Percent Inhibition
1.	Maize plant	*Bacillus subtilis*	Colonization	28–78%
2.	Maize seedling stalk	*Bacillus mojavensis*	Colonization	24–58%
3.	Seed	*Bacillus amyloliquifaciens*	Fungal growth	>82%
4.	Seed	*Microbacterium oleovarans*	Maize infection	
5.	Seed	*Enterobacter hormacchei*	Fungal growth	
6.	Maize roots	*Enterobacter cloacae*	Colonization in roots	<50%
7.	Maize stalk	*Clonostachys rosae*	Colonization	50%
8.	Rhizoplane and endorhizosphere region	*Arthrobacter globiformis*	Root colonization	69–80%
9.		*Azotobacter armeniacus*	Root colonization	56–75%

## Data Availability

Not applicable.
